# Bio-instructive hydrogel expands the paracrine potency of mesenchymal stem cells

**DOI:** 10.1088/1758-5090/ac0a32

**Published:** 2021-07-08

**Authors:** Norman M Drzeniek, Andrea Mazzocchi, Stephan Schlickeiser, Steven D Forsythe, Guido Moll, Sven Geißler, Petra Reinke, Manfred Gossen, Vijay S Gorantla, Hans-Dieter Volk, Shay Soker

**Affiliations:** 1 Berlin Institute of Health at Charité—Universitätsmedizin Berlin, BIH Center for Regenerative Therapies (BCRT), Charitéplatz 1, 10117 Berlin, Germany; 2 Berlin-Brandenburg School for Regenerative Therapies (BSRT), Charité—Universitätsmedizin Berlin, Augustenburger Platz 1, 13353 Berlin, Germany; 3 Berlin Center for Advanced Therapies (BeCAT), Charité—Universitätsmedizin Berlin, corporate member of Freie Universität Berlin and Humboldt-Universität zu Berlin, Augustenburger Platz 1, 13353 Berlin, Germany; 4 Berlin-Brandenburg Center for Regenerative Therapies (BCRT), Charité Campus Virchow-Klinikum, Augustenburger Platz 1, Berlin 13353, Germany; 5 Institute of Active Polymers, Helmholtz-Zentrum Hereon, Kantstr. 55, Teltow 14513, Germany; 6 Known Medicine Inc., 675 Arapeen Dr, Suite 103A-1, Salt Lake City, UT 84108, United States of America; 7 Wake Forest Institute for Regenerative Medicine, Wake Forest School of Medicine, Winston-Salem, NC 27101, United States of America; 8 Both authors contributed equally as senior authors.

**Keywords:** secretome, biomaterial, cell delivery, injectable hydrogel, 3D culture, collagen, hyaluronic acid, angiogenesis, mesenchymal stromal cells (MSCs), biomedical engineering, material structure, cell-material interactions, biochemical cues, biointerface, tissue engineering, regenerative medicine

## Abstract

The therapeutic efficacy of clinically applied mesenchymal stromal cells (MSCs) is limited due to their injection into harsh *in vivo* environments, resulting in the significant loss of their secretory function upon transplantation. A potential strategy for preserving their full therapeutic potential is encapsulation of MSCs in a specialized protective microenvironment, for example hydrogels. However, commonly used injectable hydrogels for cell delivery fail to provide the bio-instructive cues needed to sustain and stimulate cellular therapeutic functions. Here we introduce a customizable collagen I-hyaluronic acid (COL-HA)-based hydrogel platform for the encapsulation of MSCs. Cells encapsulated within COL-HA showed a significant expansion of their secretory profile compared to MSCs cultured in standard (2D) cell culture dishes or encapsulated in other hydrogels. Functionalization of the COL-HA backbone with thiol-modified glycoproteins such as laminin led to further changes in the paracrine profile of MSCs. In depth profiling of more than 250 proteins revealed an expanded secretion profile of proangiogenic, neuroprotective and immunomodulatory paracrine factors in COL-HA-encapsulated MSCs with a predicted augmented pro-angiogenic potential. This was confirmed by increased capillary network formation of endothelial cells stimulated by conditioned media from COL-HA-encapsulated MSCs. Our findings suggest that encapsulation of therapeutic cells in a protective COL-HA hydrogel layer provides the necessary bio-instructive cues to maintain and direct their therapeutic potential. Our customizable hydrogel combines bioactivity and clinically applicable properties such as injectability, on-demand polymerization and tissue-specific elasticity, all features that will support and improve the ability to successfully deliver functional MSCs into patients.

## 1. Introduction

Mesenchymal stromal (stem) cell (MSC) therapy is a promising regenerative treatment option for a variety of disease conditions, including but not limited to cardiovascular, neurological, musculoskeletal, immunological, and hematologic disorders [1–5]. Despite their multi-lineage differentiation potential, recent evidence challenges the initial concept that transplanted MSCs can reliably engraft and replace damaged cells and tissues. Instead, MSCs are now thought to survive only temporarily after *in vivo* application and to primarily mediate their effects through the secretion of paracrine mediators, including pro-angiogenic, neuroprotective and immunomodulatory cytokines [6–8].

Intravenous injection, the most common route of cell administration, results in clearance of MSCs from the recipient body within hours. As a consequence of this fast clearance there is a low effective cell dose at the site of injury and a short time window for paracrine effects [7, 9–11]. As such, despite encouraging pre-clinical data and over 1,000 registered clinical trials using MSCs, clinical efficacy data has not lived up to initial expectations with only a few exceptions [10, 12]. Local injection of *in vitro* expanded MSCs either directly at the site of tissue damage, or intramuscularly, can improve cell engraftment and increase the local cell dose, resulting in stronger paracrine effects. However, the efficacy of this approach is dependent on whether MSCs remain functional in the harsh environment of the tissue into which they are administered. Fortunately, this intratissue administration route opens up the opportunity to deliver cells in a defined biomaterial carrier able to physically shield cells from challenging and highly variable microenvironments, sustain their paracrine activity and guide the MSCs’ biological effects toward a specific healing scenario.

Several biomaterial strategies for cell delivery have been proposed, but most are limited by either a lack of bioactive cues that would support cell functionality or by poor usability that makes handling and local therapeutic application of the material difficult. Macroporous scaffolds are often fabricated from synthetic polymers or natural biopolymers, such as collagen, and excel in providing cell attachment sites, allowing for cell–matrix and cell–cell interactions and directing cell behavior [13–15]. Scaffold materials often undergo complex patterning methods, such as lyophilization and electrospinning. Whilst these methods provide great flexibility in generating scaffolds that can mimic microanatomical structures, they are often not possible to perform under physiological conditions and therefore can harm pre-seeded cells [16, 17]. Another major drawback to solid scaffolds is that, although they often recapitulate the native extracellular matrix (ECM) more closely than hydrogels and provide a myriad of biologically relevant cues, their application is limited to indications where surgical implantation is possible.

Aqueous hydrogels on the other hand are injectable and provide physical protection for encapsulated MSCs. They are broadly defined as polymer networks, commonly comprised of hyaluronic acid (HA), gelatin, polyethylene glycol (PEG), fibrin, alginate or polycaprolactone, which have the ability to swell in aqueous environments [18, 19]. Hydrogels can be prepared in a liquid phase and then crosslinked to a solid state with distinct mechanical properties. Hydrogel crosslinking can be achieved by different mechanisms, allowing for time- or temperature-dependent gelation and can even be triggered on demand by external stimuli such as light [20, 21]. These properties make hydrogels attractive for *in vivo* delivery of cells, where the cell suspension can be injected using a syringe or minimally invasive interventions [22]. Unfortunately, hydrogels often entrap cells in a highly hydrophilic environment that has few bioactive cues, thereby failing to support and instruct their therapeutic activity [14].

We hypothesized that increasing the presence of biologically relevant cues in an injectable hydrogel cell carrier could stimulate the secretion of therapeutically relevant mediators and that diversification of material-derived cues may further modulate the paracrine profile of encapsulated MSCs. To test this hypothesis, a biopolymer hydrogel (COL-HA) was formulated entirely from materials that are known to allow for cell–matrix interactions, such as collagen and HA, additionally functionalized with the glycoproteins laminin or fibronectin, and crosslinked through a mechanism that eliminated the need for additional bio-inert crosslinking components. The hydrogel stiffness and structure, cell viability, morphology and the paracrine profile were analyzed and compared to an industry standard gelatin-HA (Gel-HA) hydrogel. MSCs encapsulated within COL-HA formulations released different patterns of pro-regenerative factors than MSCs cultured in 2D or in other hydrogels. In depth secretome profiling predicted increased pro-angiogenic activity of COL-HA-encapsulated MSCs, which was confirmed using an assay to assess endothelial cell (EC) capillary network formation in the presence of COL-HA-encapsulated MSC conditioned media (CM).

Biomaterial-guided regulation of the MSC secretome adds important new insights to the current understanding of MSC therapy and the successful combination of usability and bio-instructive properties in COL-HA hydrogels can inform future studies into the next steps in improving methods for the successful delivery of MSC therapeutics into patients.

## 2. Materials and methods

### 2.1. Fabrication of hydrogels

**Figure 1.**
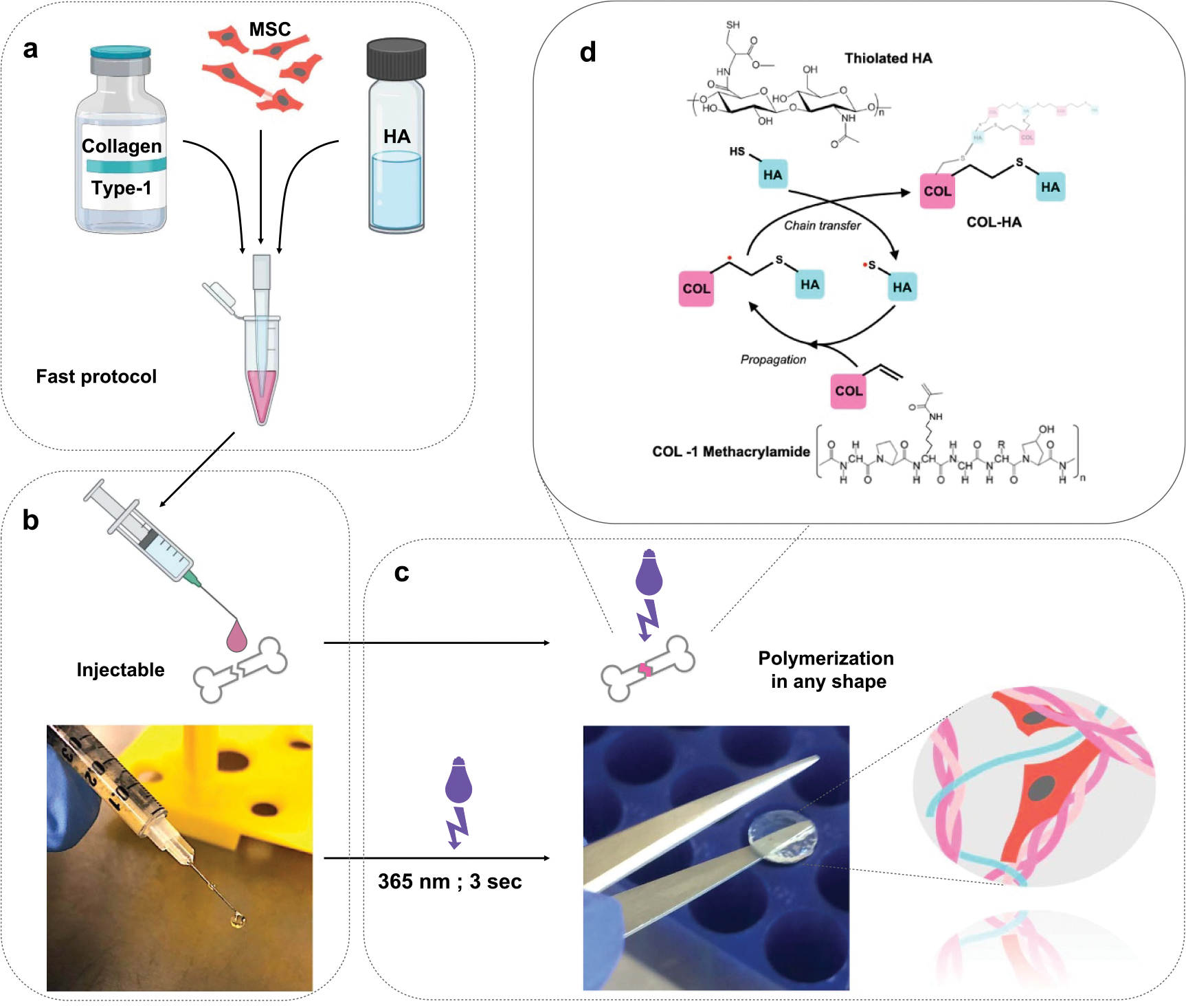
Fabrication of the COL-HA hydrogel (a) MSC are resuspended in collagen-HA hydrogel. (b) The cell-hydrogel mix can be applied using a pipette or syringe. (c) The hydrogel can be polymerized on demand with a short UVA light pulse and maintains its shape in the crosslinked state. (d) Crosslinking of the biopolymer network through radical mediated thiol-ene addition of thiolated HA to collagen methacrylamide.

Collagen-HA hydrogels (COL-HA) were prepared from telopeptide-intact methacrylated type 1 collagen (Advanced Biomatrix, Carlsbad, CA) and thiolated hyaluronic acid (Hystem; Advanced Biomatrix) and crosslinked through a light-initiated thiol-ene radical addition (figure 1). Lyophilized collagen was dissolved in 20 mM acetic acid overnight at +4 °C to a concentration of 8 mg ml^−1^. Thiolated HA was dissolved in PBS to a concentration of 10 mg ml^−1^.

For COL-HA functionalization, 1 mg ml^−1^ solutions of thiolated glycoproteins (natural mouse laminin (ThermoFisher Scientic, Waltham, MA) or human plasma fibronectin (Sigma Aldrich, St. Louis, MO)) were prepared using a protein thiolation kit (Expedeon, Over Cambridgeshire, UK) according to the manufacturer’s instructions.

Immediately before use, the collagen was brought to physiological pH and mixed with HA in a 3:1 ratio, then with laminin or fibronectin solution (or PBS) in a 2:1 ratio by volume for a final concentration of 4 mg collagen/ml, 1.67 mg HA/ml and optionally 0.33 mg ml^−1^ of glycoprotein. The liquid mix was used either for cell encapsulation or cast in a mold for photopolymerization. 0.02% (w/v) of 2-hydroxy-4′-(2-hydroxyethoxy)-2-methylpropiophenone (Sigma Aldrich, St. Louis, MO) was used to trigger the free-radical addition under exposure to UVA light of 1.4 W cm^−2^ for 3 s, resulting in instantaneous photo-initiated polymerization and hydrogel formation.

To prepare control Gel-HA gels, the Hystem^™^ kit components thiolated HA, thiolated porcine type A gelatin (Bloom 250) and Extralink, a linear 3.5 kDa PEGDA linker (Advanced Biomatrix) were dissolved in PBS to a concentration of 10 mg ml^−1^, mixed in a 2:2:1 ratio by volume and photopolymerized under the same conditions as described for COL-HA, resulting in final concentrations of gelatin and HA of 4 mg ml^−1^ each. To modulate gel stiffness, the linear PEGDA linker was replaced by aqueous solutions of 4-(8-) arm PEG acrylate (2 kDa, 10 kDa, 20 kDa MW; Creative PEGWorks, Durham, NC) at 0.5 mM (1 mM; 5 mM; 10 mM) concentrations (table 1). The four-arm 20 kDa linker at a concentration of 5 mM matched closely the stiffness of COL-HA gels and was therefore chosen as a stiffness-matched control for COL-HA (Gel-HA stiff).

### 2.2. Physical characterization of hydrogels

Pore size was measured from scanning electron microscopy (SEM) images of cell-free hydrogels. Triplicates of each hydrogel condition were prepared side by side as described above, transferred into cylindrical molds (250 *μ*l/gel) and photo-crosslinked. The gels were frozen at −80 °C overnight and lyophilized for 72 h. Dehydrated constructs were broken to expose the inner structure, sputter coated with gold/palladium particles and assessed with scanning electron microscopy at an accelerating voltage of 7.0 kV and a working distance of 5.6–8.1 mm (GeminiSEM 300, Zeiss, Oberkochen, Germany). Pore size was measured manually using ImageJ. Median values for each hydrogel condition were determined based on at least 50 measured pores and each individual value was shown in the scatter plot.

Elastic modulus (*E* = *σ*/*ε*) of the crosslinked hydrogel compositions was assessed using a uniaxial compression test using a Discovery HR-2 (TA Instruments, New Castle, DE) with an 8 mm parallel plate geometry in ambient conditions. Hydrogels were prepared as described above, the final mixture in its liquid phase was transferred into previously prepared polydimethylsiloxane (PDMS) molds and photo-crosslinked into a disc shape (⊘ = 8 mm; *V* = 100 *μ*l/disc). Compression data was collected through outputs of axial force and height displacement. To determine the elastic modulus, stress and strain (*y, x*) were plotted and the linear portion of the plot was analyzed. Stress was calculated by dividing force by surface area of the hydrogels (*σ* = *F*/*A*) and strain was calculated by change in height divided by initial height of hydrogels (*ε* = Δ*h*/*h*). Hydrogels were tested in quadruplicates (*n* = 4; for functionalized COL-HA the two variants COL-HA-Lam and COL-HA-Fn were tested in duplicate each) and average values were determined for each condition.

### 2.3. Cell culture and generation of CM

Human bone marrow (BM) mesenchymal stromal cells (MSCs) were received from the core facility ‘Cell Harvesting’ of the BIH Center for Regenerative Therapies (BCRT). The cells were derived from metaphyseal BM biopsies from two patients undergoing hip replacement at Charité—Universitätsmedizin Berlin, as previously stated [23–25]. Written informed consent was given, and ethics approval was obtained from the local ethics committee/institutional review board (IRB) of the Charité—Universitätsmedizin Berlin. All experiments in this study were performed in replicates from at least two different biological donors.

The cells were cultured in low glucose DMEM containing 10% v/v fetal calf serum (FCS), 1% v/v penicillin/streptomycin (both Biochrom AG), and 1% v/v Glutamax^™^ (Thermo Fisher Scientific Inc., Waltham, MA), passaged around 80% confluence and not used beyond passage 5.

For 3D culture MSCs were trypsinized, centrifuged and resuspended in the respective hydrogel formulations to a density of 5 × 10^6^ cells ml^−1^ per 10 *μ*l droplet and were distributed in a PDMS-precoated culture plate and photo-crosslinked as described above. Cells were allowed to interact with the biomaterial matrix for seven days. Medium was changed every other day. On day 6, constructs were washed with medium and 24 h CM were collected on day 7, centrifuged and stored at −80 °C until use for cytokine measurements. To generate stimulation medium for angiogenesis, constructs were washed with PBS instead and medium on MSC-constructs was replaced by FCS-free DMEM.

### 2.4. Quantification of MSC viability and morphology

LIVE/ DEAD viability assay (ThermoFisher Scientic, Waltham, MA) was performed on day 7 of 3D culture using calcein AM (1:2000) and ethidium homodimer-1 (1:500) in culture medium. MSC-hydrogel constructs were stained at 37 °C for 1 h before imaging on a Nikon eclipse Ti fluorescence microscope (Nikon Instruments Inc., Tokyo, Japan) or a Leica TCS LSI macro-confocal microscope (Leica, Wetzlar, Germany). Viability for each experimental group was calculated by manually counting live and dead cells. For robust results, three biological replicates were assessed for each gelatin condition, four for the functionalized COL-HA conditions and five for unfunctionalized COL-HA. Four imaged areas were analyzed from each. To quantify the observed changes in cell shape, the Analyze Particles function in FIJI was used and spindle shape was expressed as the reciprocal of circularity [26], with a value of 1 representing a perfect circle. Biological triplicates *n* = 3 were analyzed, except for COL-HA: *n* = 6.

### 2.5. Cytokine profiling

Cytokine levels in CM from cell-hydrogel constructs were analyzed using an Olink Target 96 proximity extension assay (Olink Bioscience, Uppsala, Sweden). Biological quadruplicates were tested. Briefly, CM was mixed at a 1:3 ratio with a proprietary mix of cytokine-specific antibodies labeled with DNA oligonucleotides. When two specific probes bound an analyte they formed an ampliconthat could be quantified by high-throughput RT-PCR. Details on this technology, including detection limits, validation data and reproducibility can be found on the company’s website (www.olink.com/downloads). Relative concentration values for 266 different cytokines were given as normalized protein expression (NPX) units. A marker was considered as detected if more than three samples in any group had NPX values exceeding its limit of detection (LOD). The latter was defined as the larger of either the manufacturer provided LOD or the LOD estimated from own negative controls (blank medium measurements) plus three standard deviations. To validate the proximity extension assay and obtain absolute concentrations, the cell culture experiments were repeated using new batches of CM VEGF, basic fibroblast growth factor (bFGF) and OPG were quantified using ELISA (all R&D Systems, Minneapolis, MN) according to the manufacturer’s instructions. Blank values from unconditioned DMEM were subtracted (*n* ⩾ 8 for VEGF and bFGF; *n* = 4 for OPG). Values were expressed as mean ± SEM.

### 2.6. Assessment of endothelial networks

An optimized protocol of a widely implemented endothelial tube formation assay was used to quantify pro-angiogenic effects [24, 25]. Briefly, HUVEC cells previously pre-screened for cytokine response (Promocell, Heidelberg, Germany) were starved for 10 h in serum-reduced basal endothelial medium and seeded at a density of 3 × 10^4^ cells cm^−2^ in a 48 well cell culture plate coated with Geltrex (Thermo Fisher). Cells were stimulated with biological quadruplicates of hydrogel-MSC-derived 24 h CM in a 1:5 dilution in 2.5% supplemented basal endothelial medium (Promocell) and imaged after 16 h using a fluorescence microscope (Nikon). Unconditioned MSC medium was used for a negative control. For the positive control VEGF levels were previously measured by ELISA (R&D Systems) and the highest detected VEGF levels (stimulation group COL-HA-Lam) were matched using recombinant human VEGF-A165 (#293-VE R&D Systems, Minneapolis, MN).

Bright field images of formed networks were analyzed (at least *n* = 13 per condition) and network-associated parameters were quantified using FIJI and the Angiogenesis Analyzer macro [27]. For fluorescent images, cells were stained for 30 min using calcein AM (ThermoFisher).

### 2.7. Statistical data analysis

Values are depicted as mean (± standard error of the mean (SEM)) or median. Detailed information can be found in the figure legends. Experiments were repeated independently for *n* = 4 biological replicates, unless indicated otherwise. Levels of statistical significance were set at ^∗^*p* < 0.05, ^∗∗^*p* < 0.01, ^∗∗∗^*p* < 0.001.

Statistical analysis was carried out in GraphPad Prism 8.0 (GraphPad Software Inc., USA) and R version 4.0.2. A two-tailed student’s t-test (two groups) or one-way ANOVA with Holm–Sidak’s post-hoc test (>two groups) was used as appropriate to test for significant differences between groups. Repeated measures ANOVA was carried out for quadruplicate measurements with Holm multiple comparisons adjustment using the *emmeans* package.

Heatmaps were generated with the *ComplexHeatmap* package. For clustering, a semantic distance matrix was computed using the *GOSemSim* package. Enriched gene ontology terms were derived for each cluster utilizing the *ClusterProfiler* package. Representative terms for angiogenesis, neuroprotection, immunomodulation, hemostasis and ECM remodeling were chosen and summarized for each detected cytokine under the five categories. The subterms can be found in supplementary figure 1.

## 3. Results

### 3.1. Hydrogel design and fabrication

The COL-HA hydrogels were designed as a cell delivery vehicle to modulate the paracrine effects of cells for human therapeutic application [18, 28, 29]. Collagen type 1 (COL) was chosen as the main hydrogel component because it is a highly bioactive substrate ideal for mimicking biological microenvironments, has been shown to promote MSC attachment and viability [13, 30, 31] and it is naturally enzymatically degradable [18, 28, 29, 32–34]. Native collagen crosslinks through fiber-self-assembly in a continuous, temperature-dependent process. However, these characteristics also limit the application of pure collagen as an injectable hydrogel due to lack of control over polymerization and mechanical properties.

Therefore, thiolated HA, a biodegradable glycosaminoglycan which is known to orchestrate tissue repair and angiogenesis [18, 28, 35], was used to crosslink collagen methacrylamide through a light-triggered thiol-ene reaction between the vinyl groups of the COL and sulfhydryls on HA (figure 1(d)). This eliminated the need for non-bioactive crosslinking molecules such as PEG (figure 1(a)). To diversify cell-instructive cues, the pure COL-HA formulation was functionalized with either laminin (COL-HA-Lam) or fibronectin (COL-HA-Fn; figure 1(d)). An advantage of the thiol-ene reaction, aside from its biocompatibility and bioorthogonality [21, 36, 37], is that the crosslinking can be spatially and temporally controlled with UVA light, allowing an on-demand phase transition from an injectable fluid state to a solid (form-stable) gel (figures 1(b) and (c)). This is important in the application context, where user-controlled crosslinking is beneficial for injection and precise immobilization of transplanted cells. The hydrogel design resulted in an injectable COL-HA formulation that consists exclusively of bioactive components, which can be prepared quickly using a clinician-friendly protocol and crosslinked on demand (figure 1).

### 3.2. Physical characterization of hydrogels

**Figure 2.**
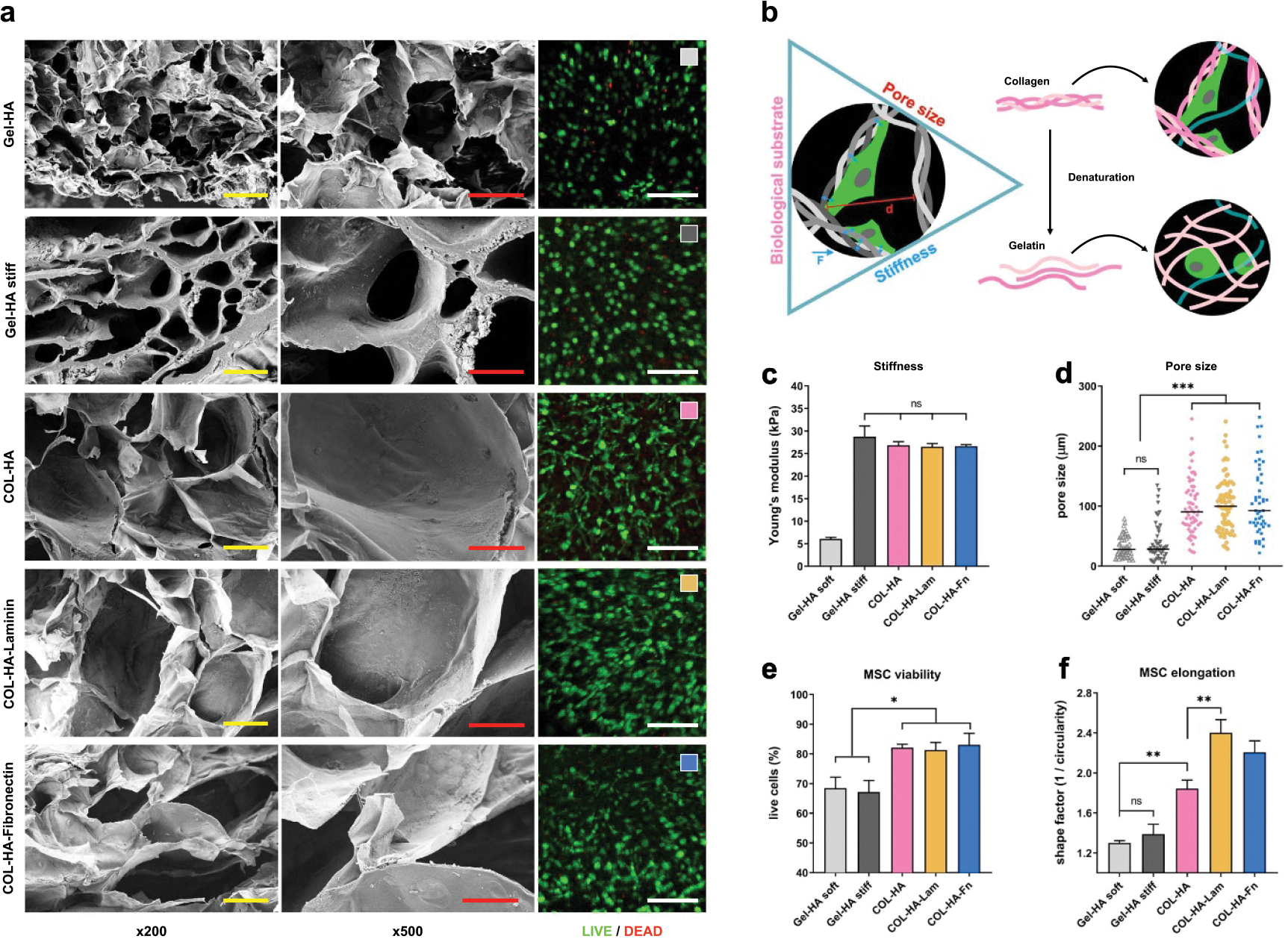
Microporous COL-HA hydrogels promote cell viability and stromal morphology of encapsulated MSCs. (a) From left to right: SEM images at two magnifications (×200; ×500) for all hydrogel conditions without cells show the inner structure of lyophilized hydrogels. Right column: representative confocal images of live/dead stained MSC at day 7 in the native hydrogel. Scale bars are 100 *μ*m, 50 *μ*m and 250 μ*m* respectively. (b)A proposed model of biological cues, structure and stiffness exerting bio-instructive effects at the cell-material interface. Gelatin is derived from collagen and shares the same amino acid sequence but differs in its biological, mechanical and structural properties. (c) Hydrogel stiffness expressed as compressive modulus. (d) Individual pore diameters (short axis) of lyophilized gels were measured from SEM images. Median pore size is shown. (e) Viability of MSC cultured in hydrogels for 7 d. (f) Elongation of cells in the hydrogels on day 7 was quantified in Image J as circularity, then expressed as its reciprocal. (*p < 0.05; **p < 0.01; ***p < 0.001; ns: *p* ⩾ 0.05; all values except (d) are mean ± SEM).

Cells in living tissues not only receive biochemical cues but are also subject to biomechanical load and themselves exert tensile and compressive forces on their surrounding matrix upon attachment (figure 2(b)). The resistance of a hydrogel to such deforming forces can be described by Young’s elastic modulus (*E*) [38, 39]. *E* of the COL-HA hydrogels and the commercially available Gel-HA control hydrogels were determined by a uniaxial compression test in their cross-linked state. Physiologically relevant values between ~26 kPa for COL-HA and ~6 kPa for Gel-HA were obtained, which are similar to the stiffness of muscle and lung or liver, respectively (figure 2(c)) [38, 39]. There was no difference in stiffness between pure COL-HA hydrogels and those functionalized with laminin or fibronectin. Since the standard formulation of Gel-HA was found to have a significantly lower *E* than COL-HA, we engineered a stiffer variant of the control hydrogel (Gel-HA stiff), which matched the *E* of COL-HA for better comparability (figure 2(c), table 1).

To characterize structural differences between hydrogel groups, the median pore diameter of lyophilized hydrogels was determined and quantified through SEM (figures 2(a) and (d)). COL-HA hydrogels were found to have larger pores than Gel-HA hydrogels. Pore size did not vary significantly between pure and functionalized COL-HA conditions. Despite the comparable median diameter, the pores of the stiff Gel-HA control were more heterogeneous and had thicker walls than the soft Gel-HA hydrogel (figure 2(a), 500× magnification).

The physical characterization of COL-HA hydrogels revealed that UV-triggered crosslinking resulted in reproducibly form-stable constructs that matched elasticity levels of native tissues, despite the crosslinker-free hydrogel design. Independently of differences in stiffness, COL-HA had larger pores in the lyophilized state than both Gel-HA controls.

### 3.3. COL-HA hydrogels promote cell viability and stromal morphology of encapsulated MSCs

To evaluate whether the different material properties affect viability and morphology of encapsulated cells, 5 × 10^6^ MSCs/ml were resuspended in each gel formulation prior to photo-crosslinking. Cells remained viable inside the hydrogels for at least a week (figure 2(a) right column). Quantification of live to dead cell ratio at day 7 post-encapsulation revealed a significantly higher cell viability of COL-HA-encapsulated MSCs (COL-HA-MSC; viability > 80%) versus Gel-HA-encapsulated MSCs (Gel-HA-MSC; viability < 70%). Viability did not vary significantly between pure and functionalized COL-HA conditions (figure 2(e)).The lowest cell viability was observed in Gel-HA stiff hydrogels.

To assess morphological differences in MSC between all conditions, confocal imaging was used (figure 2(a) right column). Circularity of living cells was quantified using automatic image analysis and the elongation was expressed as the reciprocal of circularity, where a value of 1 corresponds to a perfect circle (figure 2(f)). Independent of the Gel-HA stiffness, Gel-HA-MSCs exhibited a predominantly round morphology, whereas COL-HA-MSC developed their typical elongated fibroblastic shape. This effect was significantly enhanced by functionalization of COL-HA; MSCs in COL-HA-Lam hydrogels showed the largest cell elongation compared to all other conditions.

The results suggest that COL-HA hydrogels supported high viability of encapsulated MSCs and allowed for cell attachment and spreading within the hydrogel, resulting in a different cell morphology compared to MSCs encapsulated in Gel-HA control hydrogels.

### 3.4. COL-HA increases the paracrine activity of encapsulated MSCs

**Figure 3.**
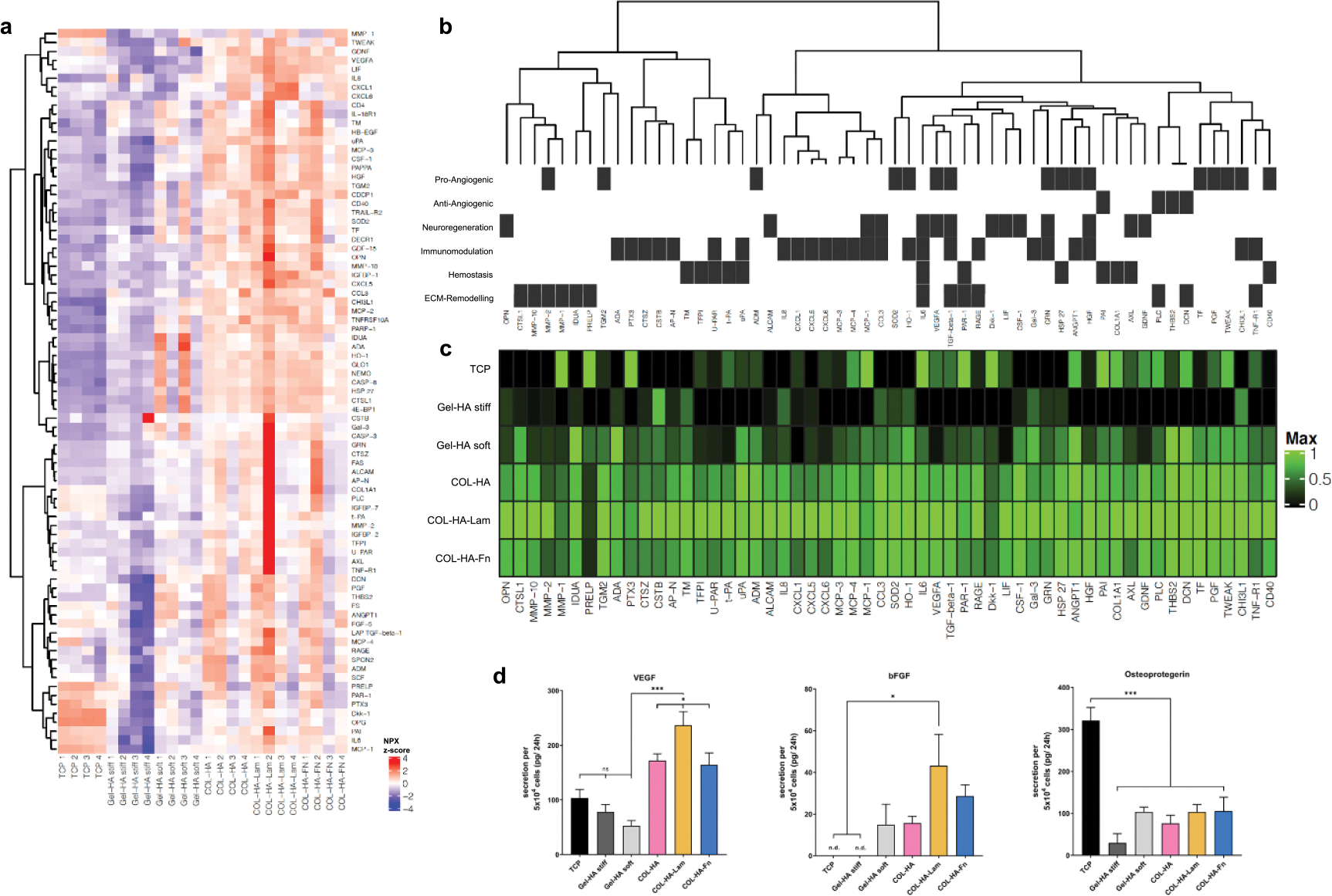
Cytokine profiling reveals expanded secretory profile of COL-HA-MSC. (a) Relative protein levels of all 81 detected analytes in CM from four biological replicates of six conditions. (b) Functional clustering of proteins relevant for angiogenesis, neuroregeneration, immunomodulation, hemostasis and ECM remodeling and (c) the detected levels of the respective proteins for each experimental group. (d) ELISA of VEGF, bFGF and osteoprotegerin. *p < 0.05; **p < 0.01; ***p < 0.001;ns: *p* ⩾ 0.05; all bar diagrams show mean ± SEM.

It is widely recognized that the therapeutic properties of MSCs are related to paracrine or trophic effects determined by their specific secretion pattern [6, 40]. To investigate the influence of the different hydrogel microenvironments on the secretion profile, the cytokine compositions of the CM of COL-HA-MSC or Gel-HA-MSC 3D constructs were characterized and compared to 2D-cultured MSCs on tissue culture plates (TCPs) using a multiplex protein array. In line with our hypothesis, this analysis revealed a generally higher cytokine secretion of MSCs encapsulated in COL-HA constructs compared to Gel-HA or 2D TCP (figures 3(a) and (c)). Despite biological differences between MSC donors, 81 out of the 266 analytes were consistently detected (figure 3(a), supplementary table S1 available online at stacks.iop.org/BF/13/045002/mmedia) at different levels, thereby forming distinct cytokine profiles for the different experimental groups. To evaluate potential biological consequences resulting from these differences, gene ontology (GO) term enrichment analysis for biological processes was performed. Out of the 81 consistently detected cytokines, 56 analytes could be assigned to at least one of the following five categories: angiogenesis, neuro-regeneration, immunomodulation, hemostasis and ECM remodeling (figures 3(b) and S1). The concentrations of half of these analytes (28 out of 56) were significantly increased in the CM of all three COL-HA-MSC groups compared to all other experimental groups (figure 3(c)). These upregulated factors included classical pro-angiogenic growth factors, such as vascular endothelial growth factor A, placental growth factor and hepatocyte growth factor, but also other proteins, such as transforming growth factor beta 1 (TGFβ1) and interleukin-8 (IL-8), which are also known to exert pro-angiogenic effects [41–45]. Neuroprotective agents like osteopontin and glial cell-derived neurotrophic factor (GDNF) were also detected at increased concentrations in COL-HA-MSC CM compared to Gel-HA-MSC and 2D TCP-MSC [46, 47]. While only minor differences were observed between the CM from COL-HA-MSC and the COL-HA-Fn-MSC, the laminin modification further increased protein secretion. This increased secretion was especially pronounced for VEGF, bFGF and leukemia inhibitory factor (LIF) (figure 3(d)). The growth factor LIF has not only neurobiological and immunomodulatory functions but also mediates tolerogenic effects of MSCs [48, 49].

The cytokine pattern of soft Gel-HA-MSC contained lower levels of most cytokines compared to the pure COL-HA condition. For example, neuroprotective factors such as LIF or GDNF were detectable only at very low concentrations, whilst VEGF levels were lowest in the CM of Gel-HA-MSC compared to all other groups (figure 3(d)). Only five factors were either more highly secreted or their secretion levels were comparable to those in the COL-HA groups. These included the anti-angiogenic matrix proteins decorin (DCN) and thrombospondin-2, and the pro-angiogenic and vasculoprotective agent angiopoietin-1 [50–52]. Encapsulation of MSCs in the stiff Gel-HA hydrogel resulted in the lowest concentrations for most detectable analytes, compared to the other 3D hydrogel conditions.

The secretion profile of MSCs cultured on 2D TCP was entirely different compared to any 3D hydrogel condition. While most analytes were only detected at low concentrations, eight out of the 56 factors were detected at the highest concentrations in the 2D culture group. These included the inflammation marker interleukin-6 (IL-6) [53], pro-apoptotic factor dickkopf-1 (Dkk-1) and the pro-thrombotic factor plasminogen activator inhibitor-1 (PAI). Dkk-1 is associated with neuron death and Alzheimer’s disease [54] and PAI regulates blood vessel formation, but is anti-angiogenic at high concentrations [55, 56]. The osteoblastic marker osteoprotegerin (OPG), which plays an important role in bone metabolism, was detected at significantly higher levels in CM from the TCP group compared to all hydrogels [57].

To validate the protein array data, VEGF and OPG, which showed very distinct regulation, were additionally measured by ELISA (figure 3(d)). The levels of bFGF were also quantified by ELISA, as it was not included in the array panel but has been described as a crucial MSC-derived trophic factor [40]. The ELISA values closely matched the results obtained from the protein array.

The comparison of paracrine profiles from MSCs exposed to different biomaterial microenvironments suggested complex biomaterial-guided regulation of the cellular secretome. In depth analysis revealed an especially strong secretion of pro-angiogenic factors from COL-HA-Lam encapsulated cells, predicting an increased pro-angiogenic potency of cells encapsulated in this cell carrier material.

### 3.5. ECs organize into capillary-like networks when stimulated with COL-HA-MSC CM

**Figure 4.**
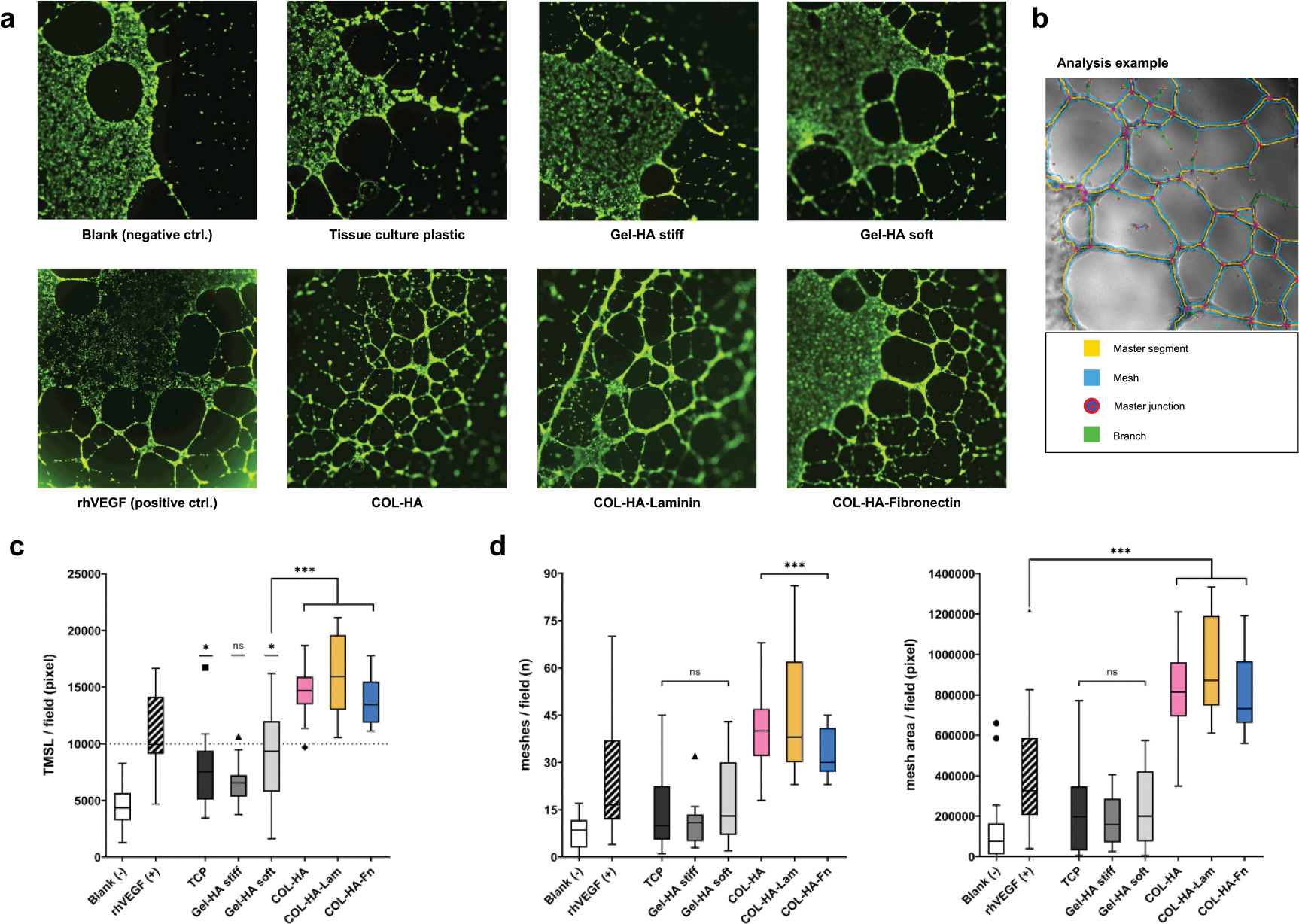
ECs organize into capillary-like networks when stimulated with pro-angiogenic CM. (a) Representative images of calcein AM stained endothelial tubes formed in response to the respective MSC/hydrogel CM indicated below. (b) Representative image of the analysis method to calculate TMSL and mesh area. To quantify the quality of formed networks, the total length of master segments (segments leading to a junction; TMSL) was quantified (c), along with the number of meshes and area covered by complete endothelial tube meshes (d) (*p < 0.05; **p < 0.01; ***p < 0.001; ns: *p* ⩾ 0.05; significance levels are relative to blank unless otherwise indicated; box plot whiskers show extreme values with Tukey’s correction for outliers).

To confirm the increased pro-angiogenic potency from cells encapsulated in COL-HA hydrogels, a validated capillary-like structure formation assay was used [24, 25]. Primary human ECs were stimulated with CM derived from each of the hydrogel-MSC groups. Recombinant human VEGF (rhVEGF) was used as a positive control at a concentration equal to the determined VEGF level of COL-HA-Lam CM. Endothelial tube formation was quantified as the total master segment length (TMSL) detectable in an imaged field (figure 4(b)). Master segments are defined as capillary fragments leading to a junction and their total length is a good parameter for the extent of endothelial tube formation under the exclusion of loose branches. Except for Gel-HA stiff, the TMSL of all other conditions was found to be significantly increased compared to the blank control (figure 4(c)). The TMSL of the three COL-HA conditions was also significantly longer than in the Gel-HA soft group.

To subsequently assess whether the MSC-hydrogel constructs were able to induce the formation of interconnected capillary-like network structures, the number of intact meshes and the total area covered by complete meshes was determined (figure 4(d)). While CM from Gel-HA constructs and from 2D TCP induced some mesh formation, most of these network structures were incomplete and did not result in a significant increase in mesh number compared to the blank control. In contrast, all COL-HA conditions as well as the rhVEGF control significantly stimulated the formation of intact endothelial meshes compared to the unstimulated wells (blank). Network formation of EC stimulated with CM from functionalized and pure COL-HA was more robust and resulted in a significantly higher mesh area compared to rhVEGF (figure 4(d)). The COL-HA-Lam biomaterial induced longer master segments and slightly larger mesh areas compared to the pure COL-HA. Interestingly, a slightly higher median mesh count and tighter meshes were observed in response to CM from pure COL-HA-MSC compared to the functionalized COL-HA hydrogels.

The biological angiogenesis readout confirmed both the functional relevance of augmented pro-angiogenic factor secretion from COL-HA-encapsulated MSCs and additionally reflected the further enhanced pro-angiogenic potency predicted for COL-HA-Lam.

## 4. Discussion

The clinical success of MSC therapy is limited by the delivery method, therefore, the aim of this study was to fabricate, characterize and optimize an application-oriented carrier material with improved bio-instructive properties for the encapsulation and delivery of therapeutic MSCs.

Two bioactive polymers, collagen and HA, were used to photo-crosslink into a hydrogel with defined, tissue-compatible mechanical properties. Both polymers are used in reconstructive or surgical indications in patients and can therefore be considered safe and clinically applicable [58–60]. In addition to the pure COL-HA formulation, two modified COL-HA variants were functionalized with either laminin (COL-HA-Lam) or fibronectin (COL-HA-Fn). Laminin is a major biological component of the basal lamina and is known to contain specific cell attachment motifs, while fibronectin is a dimer secreted from hepatocytes into the blood plasma, which plays a crucial role in blood clot formation, matrix remodeling and wound healing [61–63]. Prior studies have identified laminin as a beneficial substrate for MSC culture, capable of influencing the secretion of pro-regenerative mediators [62]. This was in line with our finding that the addition of laminin further promoted MSC elongation and secretion of angiogenic, neuroprotective and immunomodulatory mediators and induced a particularly potent pro-angiogenic MSC phenotype. However the predominant effects were seen in comparing collagen to gelatin-based materials, while the effect of glycoprotein modification was moderate in our study and could likely be blunted by glycoproteins in the serum that adsorb to the collagen matrix [63].

To put the properties of COL-HA into the context of commercially available biomaterials, an industry standard hydrogel kit, Hystem^™^ (Gel-HA), was used for comparison. Hystem^™^ has contributed to important advances in 3D cell culture and organoid development in recent years and exhibits a comparable photo-triggered phase change as COL-HA [19, 64, 65]. Gel-HA is composed of HA, PEG and gelatin, denatured collagen that shares the same amino acid sequence but has lost its tertiary triple-helical structure due to the denaturation process (figure 2(b)) [18, 28]. The loss of tertiary structure changes the availability of cell adhesion motifs on the protein surface, thereby resulting in different bio-instructive cues presented to cells by gelatin versus collagen [33, 34]. Another important difference between COL-HA and the Gel-HA control is that Gel-HA depends on a bio-inert PEG crosslinker that takes up to 20% of the hydrogel mixing volume. PEG does not allow for cells to attach, nor is it known to instruct cell behavior [66]. We eliminated the requirement for a PEG crosslinker in COL-HA since we hypothesized that a high PEG content might prevent cell–material interactions rather than provide them. Since the composition of the two Gel-HA control hydrogels differed only in the type and amount of PEG crosslinker (table 1), it is possible that the high concentration of four-arm PEG crosslinker reduced required cell–matrix interactions providing a potential explanation for the low cytokine levels in Gel-HA stiff CM.

Interestingly, structural differences were observed between collagen- and gelatin-based biomaterials (figure 2). Different studies have reported that MSCs lose their typical elongated fibroblastic shape and acquire round morphology after encapsulation in hydrogels with small pore sizes [14, 67–69]. Increased elongation of MSCs encapsulated in COL-HA, compared with poor elongation in both the soft and stiff Gel-HA conditions, suggest that the differences in cell morphology between COL-HA-MSC and Gel-HA-MSC constructs do not result from differences in bulk mechanical properties of the biomaterial, but rather depend on pore size and functionalization. The beneficial effect of large material pore size on MSC attachment and function has been demonstrated in prior studies [70]. In a setting where additional fabrication steps are added after hydrogel formation to create a porous hydrogel scaffold, differences in the material processing protocol can be used to create pores of different sizes and structures and study their effect on cells. For example, Matsiko *et al* achieved a difference in scaffold pore size by varying the lyophilization conditions between experimental groups of chemically identical scaffolds [70]. Consequently, the influence of pore size on cell function could be studied independently of the biochemical composition. In our study however, the cells were cultured in unprocessed hydrogels, while lyophilization was conducted on cell-free gels for analysis of the material structure, so that different processing protocols could not be used to fully uncouple structural parameters from biochemical cues. Because lyophilization was carried out side by side for all material groups, we assume that the observed differences in the lyophilized structure formed as a consequence of different biochemical hydrogel composition.

Encapsulation of MSCs in COL-HA demonstrated that the cells remained viable and assumed an elongated morphology within the hydrogel in comparison to commercially available hydrogels for cell culture. This observation suggested differences in interactions at the cell–material interface and we hypothesized that these differences would be reflected in the MSCs’ secretory phenotype. While most studies that investigate cell–material interactions focus only on few cell-secreted mediators, we performed a detailed analysis of biomaterial-guided changes in the paracrine profile to validate our hypothesis. This analysis revealed complex regulation patterns and predicted a potent pro-angiogenic profile for MSCs encapsulated in COL-HA. Based on our previous studies, we hypothesized that the loss of cell attachment and spreading within the biomaterial negatively affects MSC’s secretory phenotype [14]. In support of this hypothesis, the in depth secretome profiling revealed that hydrogel microenvironments differentially modulated the paracrine activity of encapsulated MSCs (figure 3). CM derived from MSCs encapsulated in COL-HA-biomaterials induced significantly stronger and more robust EC network formation compared to Gel-HA or 2D TCP (figure 4), with the strongest angiogenic effects mediated by COL-HA-Lam-MSCs. This finding validated the predicted paracrine pattern shift toward a pro-angiogenic secretome profile in a highly bioactive biomaterial microenvironment. We propose that proteomics could be used to predict the resulting biological consequences of different material fabrication strategies at an early stage and to identify new applications for proven and established biomaterials. Our results also support the concept that the complex secretome of regenerative cells, such as MSCs, has stronger therapeutic potency than the administration of a single recombinant factor, such as rhVEGF.

The differences in paracrine activity between COL-HA and Gel-HA groups could be the consequence of different cell viabilities, however, 2D TCP cultures, which are known to maintain very high cell viability, showed a limited paracrine activity in our study. Another important influence on the paracrine activity could be the hydrogel stiffness. To test this possibility, the stiff Gel-HA control was included in this study. Although the modulus of this Gel-HA stiff biomaterial matched the COL-HA groups, their secretome profiles had very little similarity. Instead, the Gel-HA stiff biomaterial resulted in the lowest cytokine levels of all experimental groups, demonstrating that higher bulk stiffness alone does not augment the paracrine activity of MSCs. Tissue-specific bulk stiffness is a relevant design parameter for future *in vivo* studies, where the injected material would need to immobilize cells in a bone fracture gap, nerve gap or contracting muscle while complying to surrounding native tissue to avoid scarring and inflammation [71, 72]. However, bulk modulus might not accurately represent mechanical forces on the microor nanoscale sensed by cells, as recent literature highlights scale dependent differences in the stiffness of living tissues [39, 73, 74]. For example, while in our study the bulk stiffness values of different hydrogel substrates can be adjusted by changing crosslinking geometry and concentration, the microscale stiffness of a single collagen molecule lies in the GPa range and differs from the microscale stiffness of gelatin or other ECM components, regardless of bulk hydrogel properties [39, 75, 76]. By consequence, despite matched bulk stiffness, different substrates could trigger different mechanotransduction events in encapsulated cells.

As a standard reference we compared the secretome of COL-HA encapsulated cells to 2D MSC culture on polystyrene TCP. Interestingly, the osteoblastic marker OPG was one of the few factors that were secreted at highest levels from MSCs cultured on TCP. MSCs are known to be able to differentiate toward osteoblastic lineages and this result could be carefully interpreted in line with studies that demonstrate stiffness dependent regulation of cell fate [77], while on the other hand the many differences between the 2D and 3D microenvironments, such as culture dimensionality, stiffness, adhesion ligand type and density make causal attributions to a single factor difficult. The 2D culture condition resulted in a completely different secretome pattern than any of the 3D hydrogel conditions, implying that cell culture on polystyrene leads to non-physiological deviations in MSC biology.

Earlier studies have provided evidence that cell–cell and cell–matrix interactions within the 3D hydrogel are crucial for the regenerative function of MSCs [14, 78]. Here, we observed that functionally relevant shifts in the pattern of paracrine activity can be achieved by modifications of the biomaterial that support these cellular interactions, such as an attachment-enabling bioactive substrate, a microporous structure and the addition of specific ligands for integrin receptors. This observation is in line with the classic concept that cells derive cues about their phenotypic identity from their surrounding ECM and that decellularized ECM extracted from specific tissues can be used to guide cell differentiation in engineered tissues or organoids [41, 79, 80]. In this study, rather than focusing on cell differentiation, we extended the concept of matrix-guided cell behavior to shifts in the secretion pattern of therapeutically relevant proteins, which we analyzed using state of the art proteomics. Specifically, within a microenvironment composed of collagen, HA and laminin we observed the strongest MSC elongation. This correlated with the high angiogenic potency of COL-HA-Lam encapsulated MSCs, as predicted by a detailed analysis of their paracrine profile and functionally confirmed using in a model of endothelial tube formation.

## 5. Conclusion

This study demonstrates the fabrication and functionality of a biopolymer hydrogel to modulate the paracrine effects of therapeutic cells. We have combined biologically relevant material cues with important application-oriented features such as injectability and controlled crosslinking to produce a bio-instructive cell carrier for MSC encapsulation and protection. The fast fabrication protocol and on demand crosslinking mechanism are scalable and clinically translatable and were designed for human application, with all individual components of COL-HA or comparable biopolymers having been previously safely implanted into patients in a clinical setting [58–60]. Microporous COL-HA hydrogels promoted viability and elongation of encapsulated MSCs, which correlated with an expanded profile of secreted regenerative mediators. This effect was especially pronounced in COL-HA functionalized with laminin, with the paracrine profile inducing a strong EC capillary network formation. These results indicate that functionally relevant shifts in the pattern of paracrine activity can be achieved by specific modifications of the biomaterial substrate and structure. Our tunable hydrogel platform creates the opportunity to generate user-defined libraries of bio-instructive hydrogels for targeted secretome modulation. In addition to the engineering of a suitable cell carrier matrix for future cell therapy studies, our study adds a new dimension to the current understanding of MSC secretome biology, suggests stronger therapeutic potency of a cell-derived secretome in comparison to the administration of a single recombinant factor and highlights important differences between 2D and 3D cell culture protocols. Our future studies will focus on *in vivo* characterization of COL-HA-encapsulated MSC in *in vivo* angiogenic and neuro-regenerative disease models as the next step towards bringing effective MSC therapies to patients.

## Supplementary Material

Supplement for Bio-instructive hydrogel expands the paracrine potency of mesenchymal stem cells

## Figures and Tables

**Table 1.** Relationship between crosslinker type and concentration and resulting hydrogel stiffness in gelatin-HA control gels: mechanical characterization of different gelatin-HA-PEG gels shows a correlation between PEG linker concentration and hydrogel stiffness. A less prominent correlation can also be observed between stiffness and the arm length of the linker.

Linker type	Linear PEGDA *	4 ARM-PEG	4 ARM-PEG	4 ARM-PEG	4 ARM-PEG	4 ARM-PEG	4 ARM-PEG*	8 ARM-PEG

Linker size (MW)	3.5 kDa	2 kDa	10 kDa	10 kDa	10 kDa	20 kDa	20 kDa	20 kDa
Linker arm length (MW/n arms)	1.75 kDa	1 kDa	2.5 kDa	2.5 kDa	2.5 kDa	5 kDa	5 kDa	2.5 kDa
Molar concentration (*μ*mol ml^−1^)	2.86 mM	5 mM	1 mM	5 mM	10 mM	0.5 mM	5 mM	0.5 mM
Mass concentration (w/v)	**1%**	**1%**	**1%**	**5%**	**10%**	**1%**	**10%**	**1%**
Hydrogel stiffness	**6069**	**6180**	**6845**	**14 048**	**22 760**	**8680**	**28 744**	**6822**
(Pa); SD	691	788	1723	1491	429	2766	4724	388

The two conditions highlighted with an asterisk (*) were used as control hydrogels in this study.

## Data Availability

All data that support the findings of this study are included within the article (and any supplementary files).
