# Supplementary material for: Bio-instructive hydrogel expands the paracrine potency of mesenchymal stem cells
Source: Biofabrication. Author manuscript; Available in PMC 2023 Mar 19. (PMC10024818; doi:10.1088/1758-5090/ac0a32)
Supplement: Supplement for Bio-instructive hydrogel expands the paracrine potency of mesenchymal stem cells [file NIHMS1870239-supplement-Supplement_for_Bio-instructive_hydrogel_expands_the_paracrine_potency_of_mesenchymal_stem_cells.docx]

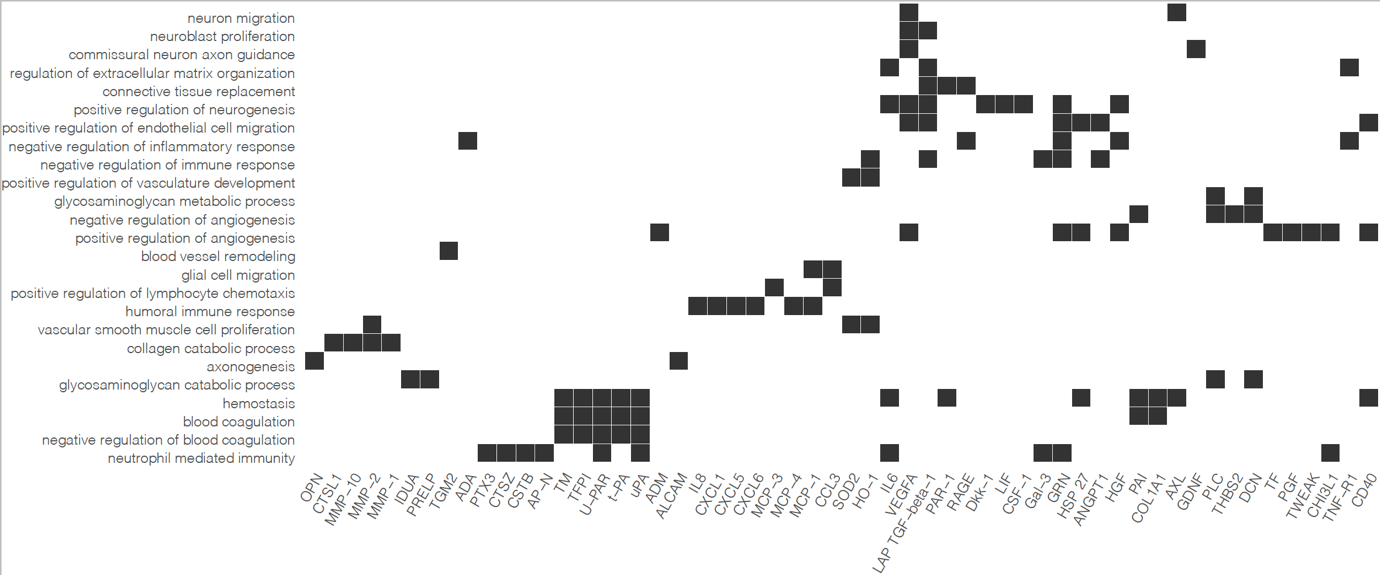


Supplementary Figure S1: Detailed gene ontology terms clustering

Original gene ontology terms assigned to cell-secreted proteins relevant in angiogenesis, neuro-regeneration, immunomodulation, hemostasis and extracellular matrix remodeling.

| BMP-6 | FALSE |
| --- | --- |
| ANGPT1 | TRUE |
| ADM | TRUE |
| CD40-L | FALSE |
| SLAMF7 | FALSE |
| PGF | TRUE |
| ADAM-TS13 | FALSE |
| BOC | FALSE |
| IL-4RA | FALSE |
| SRC | FALSE |
| IL-1ra | FALSE |
| IL6 | TRUE |
| TNFRSF10A | TRUE |
| STK4 | FALSE |
| IDUA | TRUE |
| TNFRSF11A | FALSE |
| PAR-1 | TRUE |
| TRAIL-R2 | TRUE |
| PRSS27 | FALSE |
| TIE2 | FALSE |
| TF | TRUE |
| IL1RL2 | FALSE |
| PDGF subunit B | FALSE |
| IL-27 | FALSE |
| IL-17D | FALSE |
| CXCL1 | TRUE |
| LOX-1 | FALSE |
| Gal-9 | FALSE |
| GIF | FALSE |
| SCF | TRUE |
| IL18 | FALSE |
| FGF-21 | FALSE |
| PIgR | FALSE |
| RAGE | TRUE |
| SOD2 | TRUE |
| CTRC | FALSE |
| FGF-23 | FALSE |
| SPON2 | TRUE |
| GH | FALSE |
| FS | TRUE |
| GLO1 | TRUE |
| CD84 | FALSE |
| PAPPA | TRUE |
| SERPINA12 | FALSE |
| REN | FALSE |
| DECR1 | TRUE |
| MERTK | FALSE |
| KIM1 | FALSE |
| THBS2 | TRUE |
| TM | TRUE |
| VSIG2 | FALSE |
| AMBP | FALSE |
| PRELP | TRUE |
| HO-1 | TRUE |
| XCL1 | FALSE |
| IL16 | FALSE |
| SORT1 | FALSE |
| CEACAM8 | FALSE |
| PTX3 | TRUE |
| PSGL-1 | FALSE |
| CCL17 | FALSE |
| CCL3 | TRUE |
| MMP7 | FALSE |
| IgG Fc receptor II-b | FALSE |
| ITGB1BP2 | FALSE |
| DCN | TRUE |
| Dkk-1 | TRUE |
| LPL | FALSE |
| PRSS8 | FALSE |
| AGRP | FALSE |
| HB-EGF | TRUE |
| GDF-2 | FALSE |
| FABP2 | FALSE |
| THPO | FALSE |
| MARCO | FALSE |
| GT | FALSE |
| BNP | FALSE |
| MMP12 | FALSE |
| ACE2 | FALSE |
| PD-L2 | FALSE |
| CTSL1 | TRUE |
| hOSCAR | FALSE |
| TNFRSF13B | FALSE |
| TGM2 | TRUE |
| LEP | FALSE |
| CA5A | FALSE |
| HSP 27 | TRUE |
| CD4 | TRUE |
| NEMO | TRUE |
| VEGFD | FALSE |
| PARP-1 | TRUE |
| HAOX1 | FALSE |
| TNFRSF14 | FALSE |
| LDL receptor | FALSE |
| ITGB2 | FALSE |
| IL-17RA | FALSE |
| TNF-R2 | FALSE |
| MMP-9 | FALSE |
| EPHB4 | FALSE |
| IL2-RA | FALSE |
| OPG | TRUE |
| ALCAM | TRUE |
| TFF3 | FALSE |
| SELP | FALSE |
| CSTB | TRUE |
| MCP-1 | TRUE |
| CD163 | FALSE |
| Gal-3 | TRUE |
| GRN | TRUE |
| NT-proBNP | FALSE |
| BLM hydrolase | FALSE |
| PLC | TRUE |
| LTBR | FALSE |
| Notch 3 | FALSE |
| TIMP4 | FALSE |
| CNTN1 | FALSE |
| CDH5 | FALSE |
| TLT-2 | FALSE |
| FABP4 | FALSE |
| TFPI | TRUE |
| PAI | TRUE |
| CCL24 | FALSE |
| TR | FALSE |
| TNFRSF10C | FALSE |
| GDF-15 | TRUE |
| SELE | FALSE |
| AZU1 | FALSE |
| DLK-1 | FALSE |
| SPON1 | FALSE |
| MPO | FALSE |
| CXCL16 | FALSE |
| IL-6RA | FALSE |
| RETN | FALSE |
| IGFBP-1 | TRUE |
| CHIT1 | FALSE |
| TR-AP | FALSE |
| GP6 | FALSE |
| PSP-D | FALSE |
| PI3 | FALSE |
| Ep-CAM | FALSE |
| AP-N | TRUE |
| AXL | TRUE |
| IL-1RT1 | FALSE |
| MMP-2 | TRUE |
| FAS | TRUE |
| MB | FALSE |
| TNFSF13B | FALSE |
| PRTN3 | FALSE |
| PCSK9 | FALSE |
| U-PAR | TRUE |
| OPN | TRUE |
| CTSD | FALSE |
| PGLYRP1 | FALSE |
| CPA1 | FALSE |
| JAM-A | FALSE |
| Gal-4 | FALSE |
| IL-1RT2 | FALSE |
| SHPS-1 | FALSE |
| CCL15 | FALSE |
| CASP-3 | TRUE |
| uPA | TRUE |
| CPB1 | FALSE |
| CHI3L1 | TRUE |
| ST2 | FALSE |
| t-PA | TRUE |
| SCGB3A2 | FALSE |
| EGFR | FALSE |
| IGFBP-7 | TRUE |
| CD93 | FALSE |
| IL-18BP | FALSE |
| COL1A1 | TRUE |
| PON3 | FALSE |
| CTSZ | TRUE |
| MMP-3 | FALSE |
| RARRES2 | FALSE |
| ICAM-2 | FALSE |
| KLK6 | FALSE |
| PDGF subunit A | FALSE |
| TNF-R1 | TRUE |
| IGFBP-2 | TRUE |
| vWF | FALSE |
| PECAM-1 | FALSE |
| MEPE | FALSE |
| CCL16 | FALSE |
| IL8 | TRUE |
| VEGFA | TRUE |
| CD8A | FALSE |
| MCP-3 | TRUE |
| GDNF | TRUE |
| CDCP1 | TRUE |
| CD244 | FALSE |
| IL7 | FALSE |
| OPG | TRUE |
| LAP TGF-beta-1 | TRUE |
| uPA | TRUE |
| IL6 | TRUE |
| IL-17C | FALSE |
| MCP-1 | TRUE |
| IL-17A | FALSE |
| CXCL11 | FALSE |
| AXIN1 | FALSE |
| TRAIL | FALSE |
| IL-20RA | FALSE |
| CXCL9 | FALSE |
| CST5 | FALSE |
| IL-2RB | FALSE |
| IL-1 alpha | FALSE |
| OSM | FALSE |
| IL2 | FALSE |
| CXCL1 | TRUE |
| TSLP | FALSE |
| CCL4 | FALSE |
| CD6 | FALSE |
| SCF | TRUE |
| IL18 | FALSE |
| SLAMF1 | FALSE |
| TGF-alpha | FALSE |
| MCP-4 | TRUE |
| CCL11 | FALSE |
| TNFSF14 | FALSE |
| FGF-23 | FALSE |
| IL-10RA | FALSE |
| FGF-5 | TRUE |
| MMP-1 | TRUE |
| LIF-R | FALSE |
| FGF-21 | FALSE |
| CCL19 | FALSE |
| IL-15RA | FALSE |
| IL-10RB | FALSE |
| IL-22 RA1 | FALSE |
| IL-18R1 | TRUE |
| PD-L1 | FALSE |
| Beta-NGF | FALSE |
| CXCL5 | TRUE |
| TRANCE | FALSE |
| HGF | TRUE |
| IL-12B | FALSE |
| IL-24 | FALSE |
| IL13 | FALSE |
| ARTN | FALSE |
| MMP-10 | TRUE |
| IL10 | FALSE |
| TNF | FALSE |
| CCL23 | FALSE |
| CD5 | FALSE |
| CCL3 | TRUE |
| Flt3L | FALSE |
| CXCL6 | TRUE |
| CXCL10 | FALSE |
| 4E-BP1 | TRUE |
| IL-20 | FALSE |
| SIRT2 | FALSE |
| CCL28 | FALSE |
| DNER | FALSE |
| EN-RAGE | FALSE |
| CD40 | TRUE |
| IL33 | FALSE |
| IFN-gamma | FALSE |
| FGF-19 | FALSE |
| IL4 | FALSE |
| LIF | TRUE |
| NRTN | FALSE |
| MCP-2 | TRUE |
| CASP-8 | TRUE |
| CCL25 | FALSE |
| CX3CL1 | FALSE |
| TNFRSF9 | FALSE |
| NT-3 | FALSE |
| TWEAK | TRUE |
| CCL20 | FALSE |
| ST1A1 | FALSE |
| STAMBP | FALSE |
| IL5 | FALSE |
| ADA | TRUE |
| TNFB | FALSE |
| CSF-1 | TRUE |

Supplementary Table S1: List of all measured and detected analytes

The left column includes all 266 analytes included in the protein array used in this study. The right column indicates whether the analyte was detected (TRUE) or not (FALSE).
